# Supplementary material for: Design parameters of free-form color splitters for subwavelength pixelated image sensors
Source: iScience. 2023 Sep 15;26(10):107788. doi: 10.1016/j.isci.2023.107788 (PMC10561042; doi:10.1016/j.isci.2023.107788)
Supplement: Document S1. Figures S1–S7 and Table S1 [file mmc1.pdf]

iScience, Volume *26*

Supplemental information

Design parameters of free-form color splitters

for subwavelength pixelated image sensors

Sanmun Kim, Chanhyung Park, Shinho Kim, Haejun Chung, and Min Seok Jang

Table S1. [Summary of the optimization method, design parameters, and reported efficiencies], related to Table 1 and Figure 2.

Type	Optimization Method	Period (μm)	DoF	Efficiency*	Refs. (Main)
1D RGBG	GA	1	32×4	58.3% (average), 68.2% (peak)	This work
1D NIR-RGB	Adjoint	1.6	32×40, 160×200	~80% (peak), 80-90% (peak), >99% (grayscale)	Ref. 9
Bayer 2D	Adjoint	0.64	64×64×200	>98% (peak)	Ref. 10
Bayer 2D	GA	1.6	20×20×1	49.6% (average), 60.2% (peak)	Ref. 16
Bayer 2D	GA	2	16×16×1	55.3% (peak)	Ref. 5
Bayer 2D	GA	2	20×20×1	42% (average)	Ref. 18
Bayer 2D	Adjoint	2	100×100×100	77% (peak)	Ref. 7

* The definitions of the optical efficiency are different in each work.

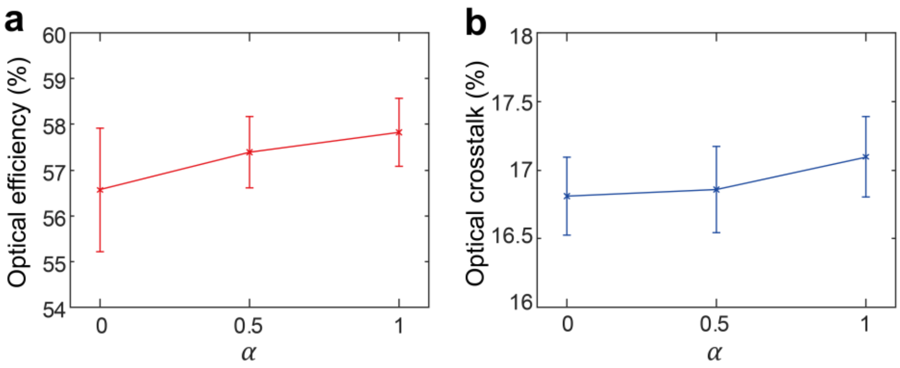

Figure S1. [Optical crosstalk], related to Figure 2. The optical efficiency and the optical crosstalk for different values of α. The symbols and the error bars represent the mean values and the standard deviations of 20 different optimization results. Here, the design objective function is chosen as in Ref. 9,

$$F(\lambda) = \sum_{i=R,G,B} \left[\alpha\eta_i(\lambda) - (1-\alpha)\left(\sum_{j \neq i} \chi_{i,j}(\lambda) \right) \right]$$

where $\eta(\lambda)$ represents the optical efficiency and $\sum_{j \neq i} \chi_{i,j}(\lambda)$ represents the optical crosstalk. We define the optical crosstalk using the electric field intensity at the focal plane. $x \in (x_{1,j}, x_{2,j})$ defines the area of the subpixel of interest.

$$\chi_{[R,G,B],j}(\lambda) = \frac{1}{2} \sum_{m=\mathrm{TE},\mathrm{TM}} \frac{\int_{x_{1,j}}^{x_{2,j}} |\mathbf{E}(\lambda,m)|^2 dx}{\int_0^P |\mathbf{E}(\lambda,m)|^2 dx} \times T(\lambda,m)$$

$\alpha \in [0,1]$ determines the priority between the optical efficiency ($\alpha = 1$) and the crosstalk ($\alpha = 0$). Although the optical crosstalk decreases with decreasing α, the amount of change is marginal. Even for $\alpha = 0$, where the optimizations were done only to minimize the optical crosstalk, the crosstalk is reduced by less than 0.5%p on average compared to the case of $\alpha = 1$. This implies that suppression of crosstalk may require either more degrees of design freedom or a wider pixel pitch.

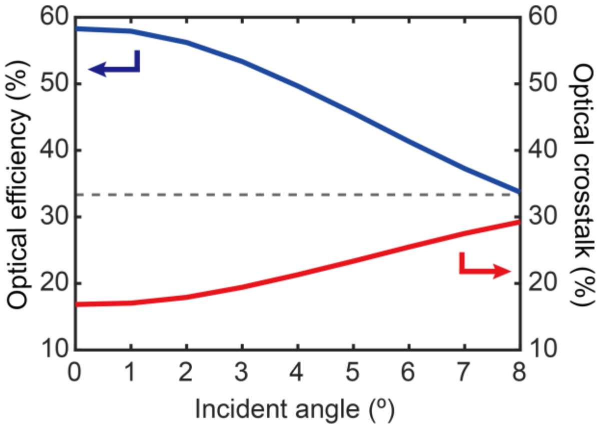

Figure S2. [Angular tolerance], related to Figure 2. The optical efficiency and the optical crosstalk as a function of incident-angle (θ). The optical efficiency drops rapidly from 58.3% to 33.7% as θ increases from 0 to 8°.

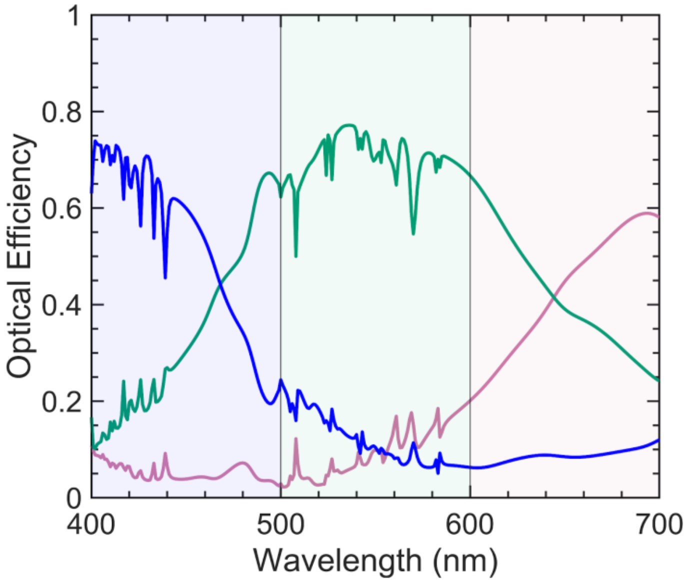

Figure S3. [The optical efficiency spectrum of RGGB configuration], related to Figure 2. The average optical efficiency and optical crosstalk of this device are 54.95% and 18.69%, respectively. The optical efficiency and crosstalk of the device in Figure 2e with RGBG arrangement are 58.29% and 16.84%. The arrangement of subpixels only has a limited effect on optical efficiency.

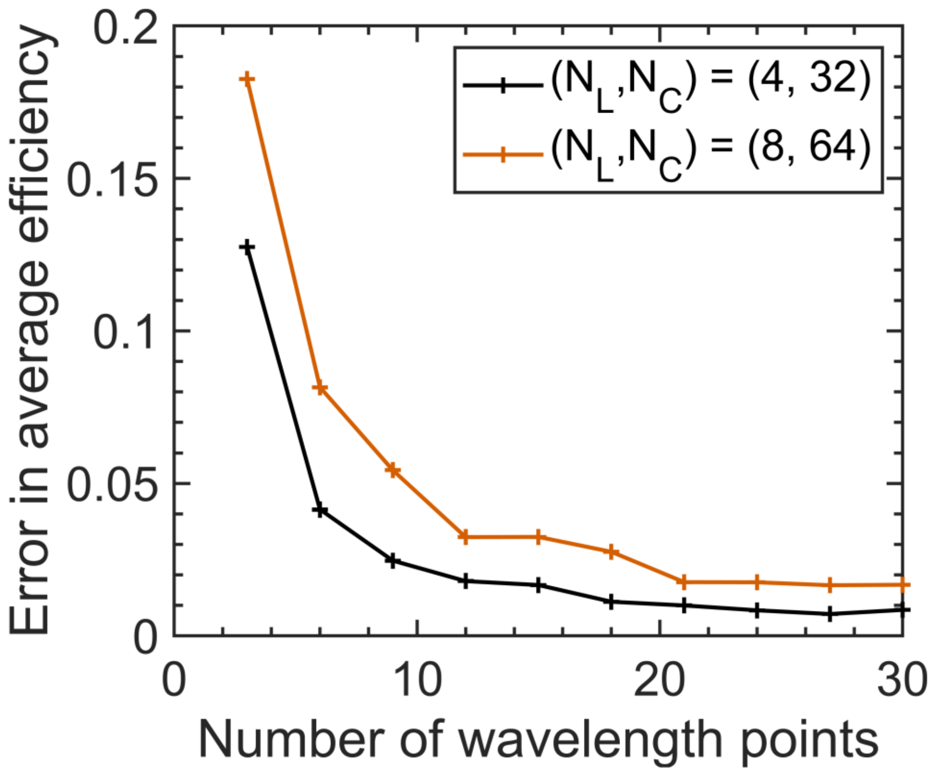

Figure S4. [Number of Sampling points], related to Table 1. The optical efficiencies reported in this work are calculated by averaging optical efficiencies over 301 wavelength points (400 nm, 401 nm, …, 700 nm). Since we are utilizing a rigorous coupled-wave analysis solver, the computational cost is proportional to the number of wavelength points. Hence, during the optimization process, the optical efficiency is sampled from thirty wavelength points (405 nm, 415 nm, …, 695 nm). The plot shows how the error in average optical efficiency changes as a function of the number of sampling points. The error in optical efficiency is defined as the discrepancy between the 301-wavelength points averaged value and the N-wavelength points averaged value. Devices with a higher DoF require more sampling points as peaks and dips are likely to be sharper for a more complex device.

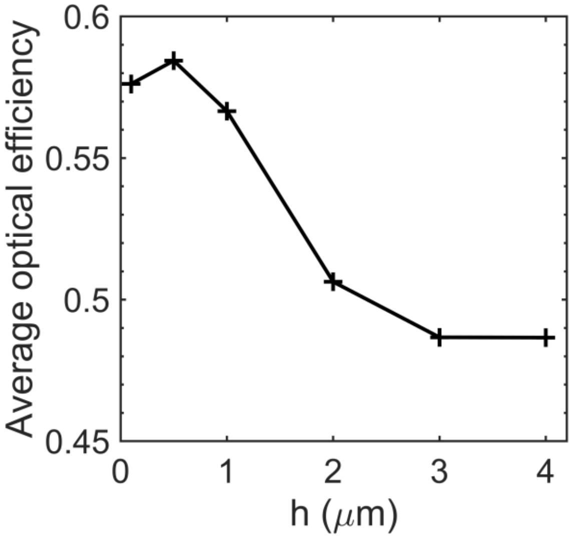

Figure S5. [Dependency of design parameter: Focal plane position], related to Table 1 and Figure 3. Optical efficiency plot as a function of focal plane position (h). The averaged optical efficiency drops with h due to the loss of near-field contribution.

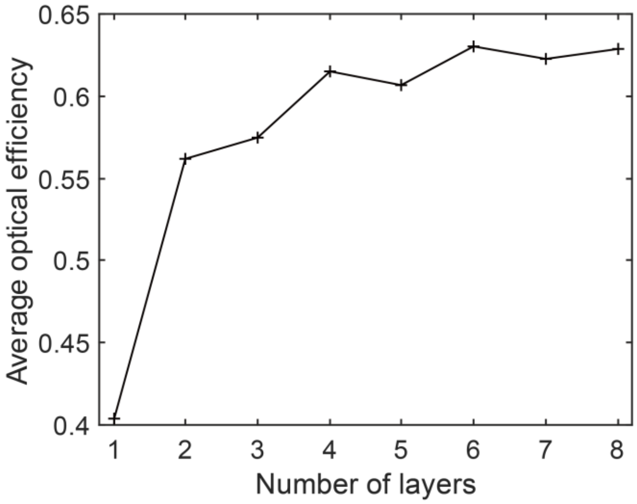

Figure S6. [Constant single layer thickness optimization], related to Table 1 and Figure 5. Optical efficiency plot as a function of the number of layers while keeping the thickness of a single layer constant at 375 nm.

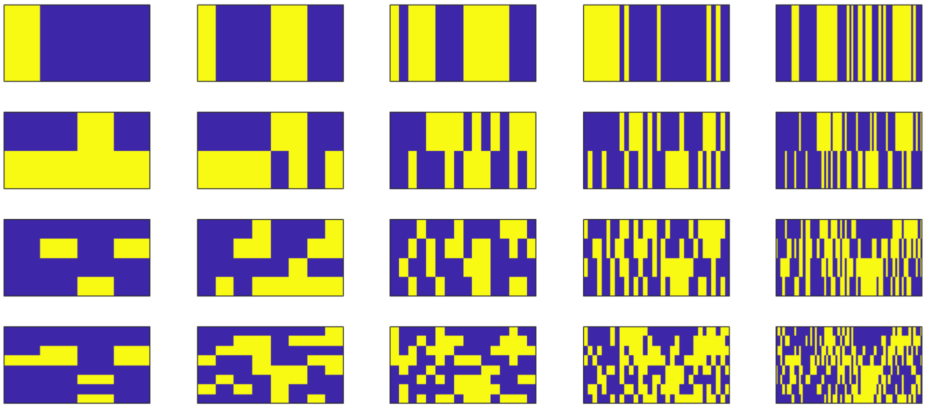

Figure S7. [Exemplary device designs for various DoF], related to Figure 5. Region colored with yellow and violet each represents space filled with n_2 and n_1, respectively.
